# The Prognostic Role of *BRAF* Mutation in Metastatic Colorectal Cancer Receiving Anti-*EGFR* Monoclonal Antibodies: A Meta-Analysis

**DOI:** 10.1371/journal.pone.0065995

**Published:** 2013-06-11

**Authors:** Zi-Xu Yuan, Xiao-Yan Wang, Qi-Yuan Qin, De-Feng Chen, Qing-Hua Zhong, Lei Wang, Jian-Ping Wang

**Affiliations:** 1 Department of Colorectal Surgery, The Sixth Affiliated Hospital of Sun Yat-Sen University, Guangzhou, People’s Republic of China; 2 Gastrointestinal Disease Institute of Sun Yat-sen University, Guangzhou, People’s Republic of China; Dartmouth, United States of America

## Abstract

**Background:**

BRAF mutation has been investigated as a prognostic factor in metastatic colorectal cancer (mCRC) undergoing anti-EGFR monoclonal antibodies (moAbs), but current results are still inconclusive. The aim of this meta-analysis was to evaluate the relationship between BRAF mutation status and the prognosis of mCRC patients treated with moAbs.

**Methods:**

Eligible studies were identified by systematically searching Pubmed, the Cochrane Library, Web of Knowledge, and OVID. Risk ratio (RR) for overall response rate (ORR), Hazard ratios (HRs) for Progression free survival (PFS) and Overall survival (OS) were extracted or calculated. Prespecified subgroup analyses were conducted in KRAS wild-type and in different study types. The source of between-trial variation was explored by sensitivity analyses. Quality assessment was conducted by the Hayden’s criteria.

**Results:**

A total of twenty one trials including 5229 patients were identified for the meta-analysis. 343 patients displayed BRAF mutations of 4616 (7.4%) patients with known BRAF status. Patients with BRAF wild-type (WT) showed decreased risks of progression and death with an improved PFS(HR 0.38, 95% confidence intervals 0.29–0.51) and an improved OS (HR 0.35 [0.29–0.42]), compared to BRAF mutant. In KRAS WT population, there were even larger PFS benefit (HR 0.29[0.19,0.43]) and larger OS benefit (HR 0.26 [0.20,0.35]) in BRAF WT. A response benefit for BRAF WT was observed (RR 0.31[0.18,0.53]) in KRAS WT patients, but not observed in unselected patients (RR 0.76 [0.43–1.33]). The results were consistent in the subgroup analysis of different study types. Heterogeneity between trials decreased in the subgroup and explained by sensitivity analysis. No publication bias of ORR, PFS and OS were detected.

**Conclusions:**

The results indicate that BRAF mutant is a predictive biomarker for poor prognosis in mCRC patients undergoing anti-EGFR MoAbs therapy, especially in KRAS WT patients. Additional large prospective trials are required to confirm the predictive role of BRAF status.

## Introduction

Colorectal cancer is the third mostly common human malignant tumor and is one major cause of cancer mortality in the western world [1]. Metastatic tumors account for 40% to 50% of newly diagnosed patients [2]. The prognosis of metastatic colorectal cancer(mCRC) remains poor. The introduction of targeted Epidermal Growth Factor Receptor (EGFR) Monoclonal Antibodies (MoAbs), namely Cetuximab and Panitumumab, has distinctly improved Overall response rate (ORR), Progression free survival (PFS) and Overall survival (OS). EGFR is a transmembrane tyrosine kinase receptor,which mediates the processes of proliferation, angiogenesis and invasion of cancer cells [3]. However, only 10%–20% of patients with mCRC can achieve benefits from anti-EGFR MoAbs [4]. EGFR expression is reported to be not correlated with clinical efficacy [5]. The benefit of targeted agents may attribute to the inhibition of its downstream signaling pathways, mainly RAS-RAF-MAPK and P3IK-PTEN-AKT [6]. Increasing evidences show that KRAS mutations at codons 12 and 13 in mCRC are predictive biomarkers of resistance to anti-EGFR MoAbs [7]. But KRAS mutations account only for 35% to 45% of nonresponders [8].

Recently, BRAF mutation (>95% of BRAF point mutations occure at BRAF V600E [9]) is introduced to be associated with resistance to targeted agents [10]. BRAF protein, a serine-threonine kinase, is the principal downstream molecular of KRAS [11]. A meta-analysis by Bokemeyer C, et al, in 2012 [12] based on two RCTs (the OPUS and CRYSTAL trials) reported that in KRAS wild-type(WT) patients, adding cetuximab to chemotherapy was beneficial for BRAF WT patients, but not for BRAF mutant patients. Another systematic review by Mao C, et al, in 2011 [13] found a response benefit for BRAF WT in KRAS WT patients, but found no response benefit for BRAF WT in unselected patients. And there is no meta-analysis for direct comparisons of PFS and OS between BRAF mutant and BRAF WT in mCRC patients using anti-EGFR MoAbs.

Here we aimed to provide a comprehensive, unbiased pooled analysis including ORR (risk ratio [RR] in patients with mutant BRAF versus(vs) these with WT BRAF) for response, PFS and OS (hazard ratios [HR] in patients with WT BRAF vs mutant BRAF) for progression and survival in patients with mCRC receiving anti-EGFR MoAbs therapies.

## Materials and Methods

### Search Strategy

We searched Pubmed, Web of Knowledge, the Cochrane library, and OVID without language limitation. The last search update was January 31, 2013. The search strategy mainly included three parts: (1) terms suggestive of “BRAF”: (ie, “BRAF” or “RAF”). (2) colorectal: (ie, “colon”, “rectal”, “colorectal”, “rectum”). (3) “cancer”: (ie, “cancer”, “carcinoma”, “neoplasm”, “tumor”, “malignan*”). Article types were restricted to clinical trials or Randomized Controlled Trials (RCT) in human. To ensure all related studies enrolled, we hand-searched several years of major journals such as ASCO (American Society of Clinical Oncology), ASCRS (American Society of Colon and Rectal Surgeons) and JCO (Journal of Clinical Oncology). The reference lists of primary studies and previous meta-analysis were scrutinized for additional publications.

The full electronic searching strategy in Pubmed was as follow: (“BRAF” or “BRAF*”) and (“colon*” or “rectal” or “colorectal” or “rectum”) and (“cancer” or “carcinom*” or “neoplas*” or “tumor” or “malignan*” or “crc”); Article types were restricted to Clinical Trial and Randomized Controlled Trial.

### Inclusion and Exclusion Criteria

The potential trials were screened for the following criteria: (1) patients with mCRC treated with cetuximab or panitumumab based therapy; (2) evalutaing BRAF mutations in the majority of patients and the number of patients with mutated BRAF was no less than one; (3) reported one or more indicators (including ORR, PFS and OS) to compare the prognosis of patients with WT BRAF to these with mutant BRAF; (4) retrospective trials, prospective trials, or randomized controlled trials. Trials evaluating progression with time to tumor progression (TTP), when TTP was defined as the time from the initiation date of Cetuximab or Panitumumab containing therapy to the first radiographic evidence of disease progression or death, were also included. We exlcuded trials without complete data, trials still in progression, and these without full text articles online. When reports overlapped or repeated, we retrived the data with longest follow up.

### Data Extraction and Definitions

Data were extracted including the first author, publication year, patient baseline characteristics, the number of patients analyzed in the study, the number of patients with known BRAF mutation, the percentage of patients with Eastern Cooperative Oncology Group (ECOG) performance status ≤1point, the proportion of liver only metastasis, study design, line of treatment, chemotherapy regimens, anti-EGFR MoAbs used and the response criteria. For clinical outcome, we collected the number of responders for calculating RRs and 95% estimation intevals for ORR. We also extracted HRs and 95% credibility intervals for PFS and OS. If separate HR was not provided, we estimated HR and its variance from published survival curves by previously described methods and models [14], [15]. Adjusted HRs and estimation intervals were also collected when reported.

Overall response included complete response (CR) and partial response (PR), non-response consisted of stable disease (SD) and progression disease (PD) according to the Response Evaluation Criteria in Solid Tumors (RECIST) [16] or World Health Organization (WHO) criteria [17]. PFS was defined as the time from the initiation date of anti-EGFR moAbs therapy to first evidence of disease progression or death of any cause, OS was defined as the time from the initiation date of anti-EGFR moAbs therapy to death of any cause. Outcome data were extracted separately in unselected population and in KRAS WT population. All data above were extracted by two independent investigators. When discrepancies existed, discussions were made to reach a consensus.

### Assessment of Study Quality

For assessing the risk of bias in individual study, we used the Hayden's criteria to assess the quality [18]. This is based on six domains of potential study biases which should be included in a review of prognostic studies: study participation, study attrition, measurement of prognostic factors, measurement of confounding variables, mesurement of outcomes, and analysis methods. The criteria is not scored, but we designed a scoring scale based on the Hayden’s criteria with some modifications to this study to quantize the assessment. The maximum score for each item was 2. Studies scoring 10–12 were defined as high quality, while these scoring 0–9 were considered low quality, just as previously defined by Maan ZN, et al. [19] (Table S1).

### Statistical Analysis

We described statistics for baseline characteristics across eligible studies. A Risk ratio (RR) of ORR was calculated by the formula 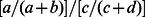 (a, b represented for the numbers of responders and nonresponders in BRAF mutant; c,d represented for the numbers of responders and nonresponders in BRAF WT in the same arm ) [20]. A HR and its variance were used directly if the trials provided. If not appropriate for direct analysis, we converted a HR and variance according to previous reported methods [14], [15]. When not reported, a HR was estimated indirectly from other statistics such as log rank p value or calculated from published Kaplan-Meier survival curves by methods and models previously mentioned [14], [15], [21]. A RR<1 for response (BRAF mutation vs BRAF WT), and HRs<1 for PFS and OS (BRAF WT vs BRAF mutation) revealed poorer pognosis of patients with mutated BRAF over these with WT BRAF in anti-EGFR treatments.

Between-trial heterogeneity was assessed by both Q^2^ statistic and I^2^ statistic for more reliability. For Q^2^ statistic, significant heterogeneity existed when p value was less than 0.10 [22]. For I^2^ statistic, values above 50% were deemed to suggest large heterogeneity; values between 25%–50% indicated modest heterogeneity; values below 25% meant low heterogeneity [23]. But the values could be largely uncertain when few trials were pooled. If the results of Q^2^ statistic and I^2^ statistic were conflicted, the conclusion of I^2^ statistic was adpoted. The effect sizes across trials, namely pooled HRs and RRs, were estimated using the fixed-effect model by Mantel-Haenszel method when no significant heterogeneity existed(X^2^ test: p≥0.10). A random-effect model by Dersimonian and Laird method was adopted when there was a noted heterogeneity (X^2^ test: p<0.10) [22], [24]. The source of heterogeneity was explored by sensitivity analysis when large heterogeneity was presented. All p values reported were two-sided.

Publication bias were assessed by Egger’s test (P<0.05 represented existing publication bias) and were reflected by visual symmetry of Begg’s funnel plot on the natural logarithm of RRs or HRs [25].

Prespecified subgroup analysis was conducted in KRAS WT patients, as increasing evidence suggested KRAS mutation to be a predictor for resistance to anti-EGFR MoAbs therapy [26]. Subgroup analysis was also conducted according to different study types such as retrospective, prospective trials and RCT in both unselected population and KRAS population, to explore whether the results of meta-analysis from different study types being consistent. Sensitivity analyses were performed to evaluate the stability of pooled results by deleting one trial each time. The source of heterogeneity was also explored when strong heterogeneity between-trial existed. All the statistical analyses in the meta-analysis were performed with STATA software, version 11.0 (Stata Corporation, College Station, TX, USA, http://www.stata.com).

## Results

### Study Selection and Characteristics

**Figure 1:**
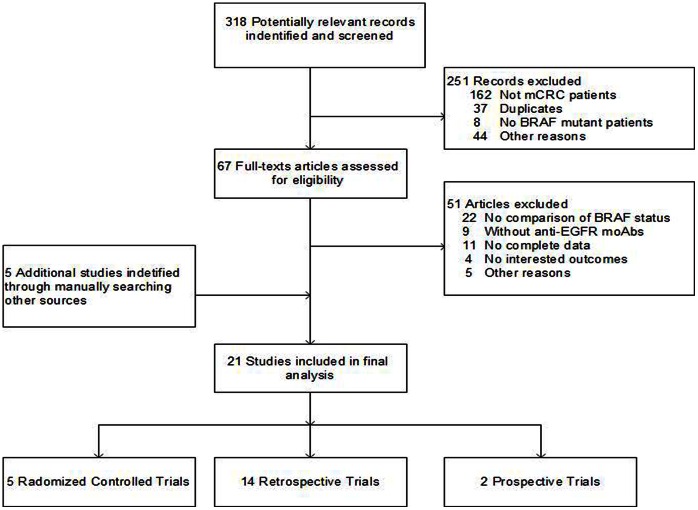
Flow Diagram for included and excluded studies.

Total 318 potentially relevant records for retrieval were identified from Pubmed (n = 55), Web of Knowledge (n = 32), the Cochrane Library (n = 14), and OVID (n = 217). After reading headings and abstracts, 251 records were excluded. The remaining 67 full-texts articles were assessed for eligibility. We excluded 51 studies which did not meet eligibility criteria. Five additional trials were identified by manually searching the preference lists of previous meta-analysis, major meetings, primary studies and major journals. Finally, 21 eligible trials were included into the meta-analysis (Figure 1).

As for the line of chemotherapy, six trials [27]–[32] used anti-EGFR MoAbs therapy as the first line, eight trials [33]–[40] as ≥second line (second or higher line), three trials [41]–[43] as ≥first line (including first, second and higher in the same trial), while four trials [44]–[47] did not report the line of treatment. Fifteen trials [27]–[33], [35]–[40], [44], [45] used cetuximab based therapies. One trial [43] used Panitumumab based theapy,while five trials [34], [41], [42], [46], [47] applied Cetuximab and Panitumumab in the same trial. Fifteen trials [28], [31], [32], [34]–[42], [44], [45], [47] evaluated response according to RECIST criteria, three trials [27], [29], [30] according to WHO criteria, two trials [33], [43] according to RECIST or WHO criteria in the same trial, while one trial [46] did not report response criteria. The 21 trials compared different types of eligible regimens. Four trials [27]–[30] involved two arms in each trial, one trial [32] involved three arms. The most commonly applied regimen was Cetuximab plus Irinotecan (in eleven trials). For quality assessment, ten studies [27]–[34], [44], [45] were within high quality scoring from 10 to 12, while eleven studies [35]–[43], [46], [47] were within low quality scoring from 7–9 (Table 1).

**Table 1:** Trial characteristics.

First author(year)	Anti-EGFR MoAbs	Chemotherapy regimens	Line oftreatment	Response criteria	Quality score
Bokemeyer(2011)	C	Arm1:FOLFOX-4;Arm2: FOLFOX-4+C	1st and ≥2nd	WHO	11
De Roock(2000)	C	C+chemo	≥2nd	RECIST or WHO	12
Di Nicolantonio(2008)	C or P	C alone,or C+IRI,or P alone	≥2nd	RECIST	11
Fornaro(2011)	C	C+IRI	≥2nd	RECIST	8
Modest(2010)	C	ArmA:C+CAPIRI; ArmB:C+CAPOX	1st	RECIST	10
Tol(2010)	C	ArmA:C+CAP+OX+Beva; ArmB: CAP+OX+Bev	1st	WHO	11
Van Cutsem(2011)	C	Arm1:FOLFIRI; Arm2: FOLFIRI+C	1st	WHO	10
Laurent-Puig(2009)	C	C+IRI, or C+FOLFIRI, or C alone	≥2nd^*^	RECIST	9
Park(2011)	C	C+OX, or C+IRI,or C+5-FU,or C alone	≥2nd^#^	RECIST	9
Saridaki(2011)	C	C+IRI, or C+OX	NR	RECIST	11
Wong(2011)	C	C+CAP+OX+Bev	1st	RECIST	11
Tveit(2011)	C	ArmA:FLOX;ArmB:FLOX+C; ArmC:FLOX(intermittent)+C	1st	RECIST	11
Loupakis(2009)	C	C+IRI	NR	RECIST	10
Spindler(2011)	C	C+IRI	3rd	RECIST	9
Cappuzzo(2008)	C	C+IRI, or C+OX, or C alone	≥2nd	RECIST	8
Sartore-Bianchi(2009)	C or P	C+chemo	NR	NR	8
Perrone(2009)	C	C+IRI	≥2nd	RECIST	9
Moroni(2005)	C or P	C+chemo, or P+chemo	1st and ≥2nd	RECIST	9
Benvenuti(2007)	C or P	C alone,or P alone,or C+IRI-based chemo	1st and ≥2nd	RECIST	7
Freeman(2008)	P	P alone	1st and ≥2nd	RECIST or WHO	7
Molinari(2009)	C or P	C+IRI, or P+IRI	NR	RECIST	8

Abbreviations: C, Cetuximab; P, Panitumumab; IRI, irinotecan; OX, oxaliplatin; CAP, capecitabine; 5-Fu, 5-fluorouracil; Bev, bevacizumab; FOLFOX-4, folinic acid+5-Fu+OX; FOLFIRI, folinic acid+5-FU+IRI; CAPOX, CAP+ OX; CAPIRI, CAP+ IRI; FLOX, folinic acid+OX; chemo, chemotherapy.

NR, Not reported; RECIST, the Response Evaluation Criteria in Solid Tumors; WHO, World Health Organization.

1st, first line treatment, ≥2nd, second or or higher line treatment; 3rd, third line treatment.

A* represented all patients but one received moAbs as a second or higher line treatment.

A # represented all patients but three received moAbs as second or higher line treatment.

Of these trials enrolled, fourteen trials [33]–[37], [39]–[47] were retrospective trials. Five trials [27]–[30], [32] were RCTs. Two trials [31], [38] were prospective trials (Table 2). A total of 5226 patients were analyzed. BRAF status was available among 4616 patients (88.3% of the total analyzed). 343 patients (7.4% of the patients with known BRAF status) displayed BRAF mutations. The mutated site was mostly V600E mutation at the extron 15 of BRAF gene. Around 60% of patients enrolled were men and median age were 61–73 years across trials. The majority of patients had good performance status with the proportion of ECOG/WHO score 0–1 point being more than 90%. Five trials [27], [28], [30], [32], [35] reported metastases confined to the liver only and the average percentage of liver only metastases was 24% (Table 2).

**Table 2:** Main characteristics of studies included.

First author, year	Study design	Patientsanalysed (n)	Patients with BRAF mutation/known BRAF status (% BRAF mutation)	Median age(range)	Sex(%, male)	Liver only metastases(%)	Performance status 0–1(%)
Bokemeyer,2011	RCT	337	11/309(4%)	61(24–82)	54%	26%	91%
De Roock,2010	Retrospective	773	36/761(5%)	61(22–86)	58%	NR	NR
Di Nicolantonio,2008	Retrospective	113	11/79(11%)	63(26–85)	71%	NR	NR
Fornaro,2011	Retrospective	54	7/52(13%)	73(70–82)	63%	13%	96%
Modest,2010	RCT	146	17/146(12%)	62(32–77)	72%	40%	NR
Tol,2010	RCT	559	45/518(9%)	62(NR)	57%	NR	NR
Van Cutsem,2011	RCT	1198	60/999(6%)	61(19–84)	61%	21%	97%
Laurent-puig,2009	Retrospective	173	5/171(3%)	NR	NR	NR	NR
Park,2011	Retrospective	75	5/71(7%)	59(29–78)	63%	NR	NR
Saridaki,2011	Retrospective	112	8/112(7%)	66(23–83)	60%	NR	NR
Wong,2011	Prospective	30	3/29(10%)	56(33–77)	53%	NR	100%
Tveit,2011	RCT	566	55/457(12%)	62(24–75)	59%	19%	96%
Loupakis,2009	Retrospective	87	13/87(15%)	66(41–79)	60%	NR	95%
Spindler,2011	Prospective	107	3/94(3%)	62(38–82)	54%	NR	90%
Cappuzzo,2008	Retrospective	85	4/79(5%)	63(29–79)	64%	NR	97%
Sartore-Bianchi,2009	Retrospective	132	11/132(8%)	64(26–85)	65%	NR	NR
Perrone,2009	Retrospective	32	3/31(10%)	57(36–78)	63%	NR	NR
Moroni,2005	Retrospective	31	1/30(3%)	66(41–85)	71%	NR	100%
Benvenuti,2007	Retrospective	48	6/48(13%)	62(39–84)	63%	NR	NR
Freeman,2008	Retrospective	62	4/62(7%)	62(29–85)	60%	NR	100%
Molinari,2009	Retrospective	38	2/36(6%)	NR	63%	NR	NR

Abbreviations: NR, not reported; RCT, randomized control trial.

Eighteen trials of 21 trials reported the numbers of responses: ten trials [28], [32], [35], [38]–[43], [47] reported responses in unselected population, seven trials [27], [30], [33], [34], [36], [37], [45] reported responses in KRAS WT population, one trial [46] reported in both population. The remaining three trials [29], [31], [44] did not report the number of responses. As for HRs and estimation intervals of PFS and OS in all 21 trials, three trials [33], [43], [46] reported HRs and estimation intervals in both unselected patients and KRAS WT patients, while two trials [31], [32] reported only in unselected population. Four trials [27], [30], [35], [45] did not convert HRs as we wanted. Four trials [29], [34], [36], [37] provided survival curves available for separately calculating HRs and variances for PFS and OS, to compare BRAF WT to BRAF mutant patients. Eight studies [28], [38]–[43], [47] did not have sufficient data on HRs and estimation intervals for PFS and OS. (Table 3).

**Table 3:** Main treatment effects and progression of patients with mCRC.

First author	ORR(responders/total patients with BRAF wt or mt treated with moAbs)				PFS		OS	
	Unselected patients		KRAS wt patients		HR and 95% CI (BRAF mt vs wt)		HR and 95% CI (BRAF mt vs wt)	
	BRAF mt	BRAF wt	BRAF mt	BRAF wt	Unselected patients	KRAS wt patients	Unselected patiens	KRAS wt patients
Bokemeyer	NR	NR	Arm1∶0/5	Arm1∶33/92	NR	NC	NR	NC
			Arm2∶2/6	Arm2∶43/72				
De Roock	NR	NR	2/24	124/326	3.82(2.38,6.12) *	3.74(2.44–5.75) *	2.93(1.85–4.65) *	3.03(1.98–4.63) *
Di Nicolantonio	NR	NR	0/11	22/68	NR	NR	NR	NR
Fornaro	0/7	10/45	NR	NR	NC	NR	NC	NR
Modest D.P	8/14	61/109	NR	NR	NR	NR	NR	NR
Tol Jolien	NR	NR	NR	NR	NR	NR	NR	NR
Van CutsemE	NR	NR	Arm1∶5/33	Arm1∶123/289	NC	NC	NC	NC
			Arm2∶5/36	Arm2∶169/277				
Laurent-Puig	NR	NR	0/5	52/110	NR	NR	NR	NR
Park	NR	NR	0/5	8/34	NR	NR	NR	NR
Saridaki	NR	NR	NR	NR	5.1(2.8–9.6) *	9.5(3.9–23.3) *	3.0(1.3–6.6) *	4.6(2.1–10.0) *
Wong	NR	NR	NR	NR	16.3(2.6–109.4) *	NR	8.3(1.9–37.4) *	NR
Tveit	Arm(B+C)	Arm(B+C)	NR	NR	2.08(1.56–2.29) *	NR	2.89(2.12–3.95) *	NR
	:7/36	:139/275						
Loupakis	NR	NR	0/13	24/74	NC	NR	NC	NR
Spindler	0/3	18/90	NR	NR	NR	NR	NR	NR
Cappuzzo	0/4	13/75	NR	NR	NR	NR	NR	NR
Sartore Bianchi	0/11	26/121	0/11	24/84	1.39(0.52–3.69) *	2.03(0.66–6.28) *	2.31(0.87–6.13) *	3.75(1.29–10.90) *
Perrone	2/3	8/28	NR	NR	NR	NR	NR	NR
Moroni	0/1	10/29	NR	NR	NR	NR	NR	NR
Benvenuti	0/6	11/41	NR	NR	NR	NR	NR	NR
Freeman	1/4	3/58	NR	NR	NR	NR	NR	NR
Molinari	0/2	2/10	NR	NR	NR	NR	NR	NR

Abbreviations: NR, not reported; NC, not converted; ORR, overall response rate; PFS, progression free survival; OS, overall survival; HR, hazard ratio; KRAS wt, KRAS wild type; CI, credibility interval; BRAF mt, BRAF mutation; BRAF wt, BRAF wild-type; moAbs, monoclonal antibodies.

Arm1 and Arm2, ArmA and Arm(B+C) represented different treatment regimens in the same trial.

A * represented adjusted HRs by Cox regression model.

### Meta-analysis

**Figure 2:**
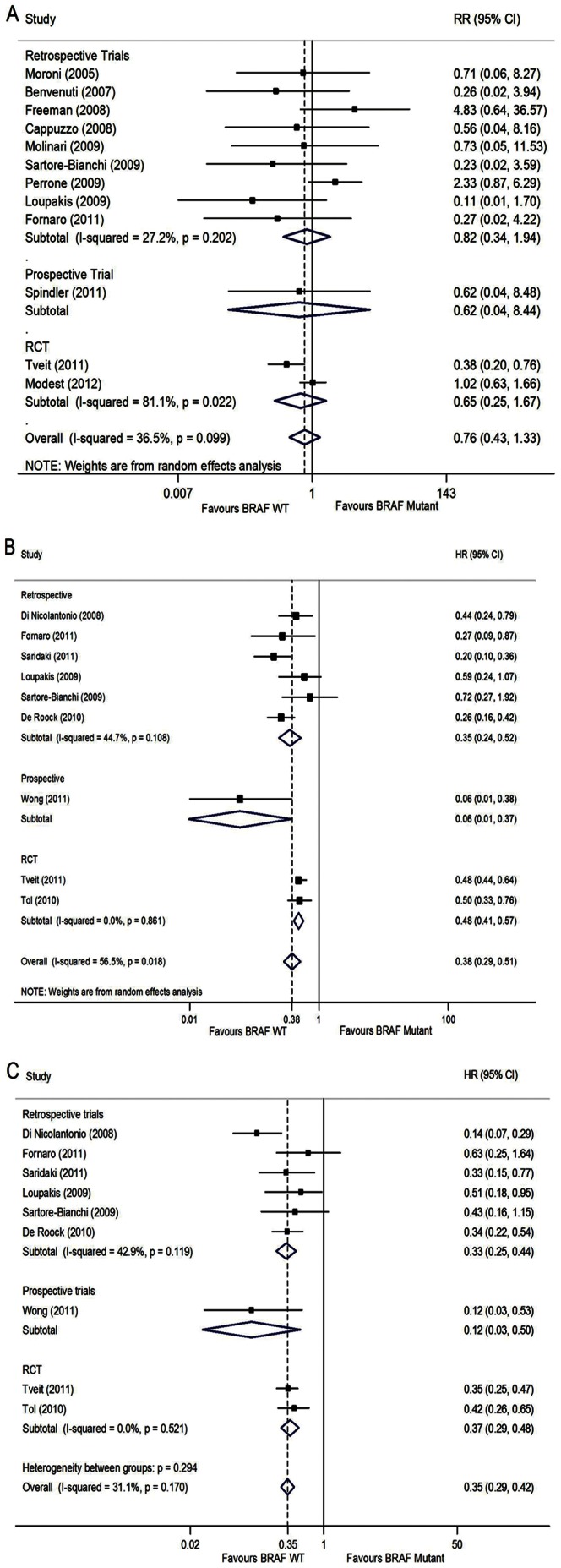
Forest plots of ORR, PFS, and OS in unselected patients in metastatic colorectal cancer. (A) RR for overall response rate (BRAF Mutant vs BRAF WT), random-effects model; (B) HR for progression free survival (BRAF WT vs BRAF Mutant), random-effects model; (C) HR for overall survival (BRAF WT vs BRAF Mutant), fixed-effects model.

We performed three different meta-analyses (namely RR for ORR, HRs for PFS and OS) upon unselected patients, KRAS WT patients only, and different study types. Larger benefits were observed for BRAF WT patients, with an improved PFS (HR 0.38, 95%CI [0.29–0.51], p<0.001, Figure 2B), albeit with differences across trials (Heterogeneity p = 0.018, I^2^ = 56.5%, random effect model, Figure 2B), comparing to BRAF mutant patients. In subgroup analysis of different study types, variation decreased to below 50% (Heterogeneity p = 0.108, I^2^ = 44.7%, random effect model, Figure 2B) across retrospective trials. There was also enough evidence of an improved OS for WT BRAF patients (HR 0.35, [0.29–0.42], p<0.001, Figure 2C) with no significant differences between trials (Heterogeneity p = 0.170, I^2^ = 31.1%, fixed effect model, Figure 2C), comparing to BRAF mutation. But difference for ORR (RR 0.76, [0.43,1.33], p = 0.328) was not significant when comparing BRAF mutant to BRAF wild-type, with no significant heterogeneity across trials (Heterogeneity p = 0.099, I^2^ = 36.5%, random effect model, Figure 2A). No publication bias were found in the three pooled analysis above by Egger’s test (ORR: p = 0.481, PFS: p = 0.185; OS: p = 0.691). Begg’s Funnel plots of ORR, PFS, and OS were listed as Figure S1, Figure S2 and Figure S3.

In the subgroup analysis of different study types in unselected population, we performed meta-analysis separately according to retrospective, prospective trials and RCT. And the results were mostly consistent with the overall findings.There were still benefits for BRAF WT patients on PFS in no matter retrospective trials (HR 0.35, [0.24–0.52], p<0.001, Figure 2B), prospective trial(HR 0.06, [0.01–0.37], p = 0.002, Figure 2B), or RCT (HR 0.48, [0.41–0.57], p<0.001, Figure 2B); and benefits for BRAF WT patients on OS in retrospective trials (HR 0.33, [0.25–0.44], p<0.001) with no significant variation (Heterogeneity p = 0.119, I^2^ = 42.9%, fixed effect model, Figure 2C), prospective trial (HR 0.12, [0.03–0.50], Figure 2C), and RCT (HR 0.37, [0.29–0.48], p<0.001, Figure 2C). We still found no evidences of improvements for BRAF WT patients on ORR in no matter retrospective trials (RR 0.82 [0.34–1.94], p = 0.647) with no significant variation (Heterogeneity p = 0.202, I^2^ = 27.2%, Figure 2A), prospective trial (RR 0.62, [0.04–8.44], Figure 2A), or RCT (RR 0.65, [0.25–1.67], p = 0.368, Figure 2A) with significant heterogeneity (p = 0.022, I^2^ = 81.1%, Figure 2A) (only two RCTs were included into the meta-analysis, sensitivity analysis to explore the the source of heterogeneity between studies was listed in Table S2).

**Figure 3:**
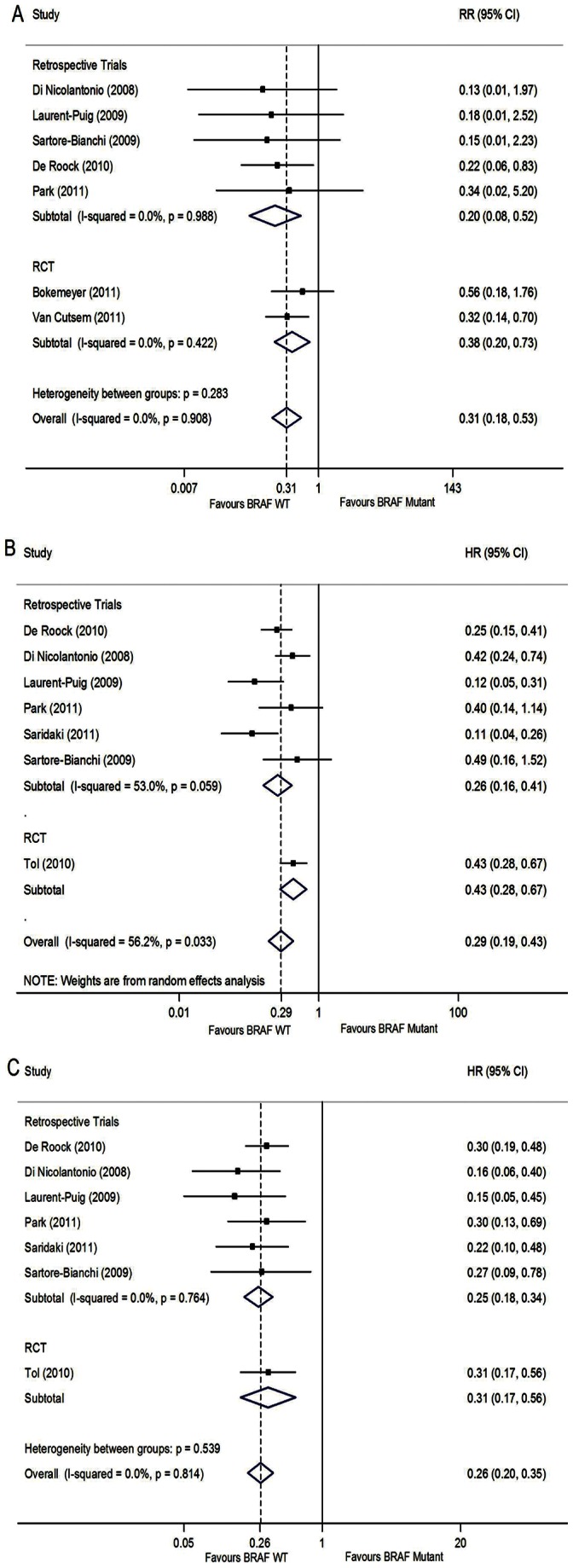
Forest plots of ORR, PFS and OS in KRAS Wild-type patients. (A) RR for overall response rate (BRAF Mutant vs BRAF WT), fixed-effects model; (B) HR for progression free survival (BRAF WT vs BRAF Mutant), random-effects model; (C) HR for overall survival (BRAF WT vs BRAF Mutant), fixed-effects model.

We then performed meta-analysis of ORR, PFS and OS seperately in KRAS WT patients. There were a PFS benefit in BRAF WT (HR 0.29, [0.19–0.43], p<0.001), although there were considerable differences between the trials (Heterogeneity p = 0.033, I^2^ = 56.2%, random effect model, Figure 3B). In the subgroup analysis of retrospective trials, heterogeneity decreased to still above 50% (p = 0.059, I^2^ = 53.0%, Figure 3B). In sensitivity analysis, we tried to explore the source of heterogeneity from study quality, age and sex, but we didn’t find out the source. Heterogeneity across trials may come from others (Table S2). We also conducted sensitivity analysis by deleting one study each time and the results were still consistent (results not provided in the study), which revealed the stability of the conclusion.There was also evidence of an OS benefit in BRAF WT patients (HR 0.26, [0.20–0.35], p<0.001 ) (BRAF WT vs BRAF mutant) without any suggestion of variation across trials (Heterogeneity p = 0.814, I^2^ = 0.0%, fixed effect model, Figure 3C), and there was also an ORR benefit in BRAF WT patients (RR 0.31 [0.18–0.53], p<0.001) (BRAF mutant vs BRAF WT), without considerable variation across studies (Heterogeneity p = 0.908, I^2^ = 0.0%, fixed effect model, Figure 3A). No publication bias existed in pooled analysis above by Egger’s test (PFS: p = 0.368; OS: p = 0.071; ORR: p = 0.219) (Begg’s Funnel plots were not posted in the article).

In the subgroup analysis of different study types in KRAS WT patients, the findings of retrospective trials and RCT were mostly similar to the overall findings. Significant improvements on PFS for BRAF WT patients in both retrospective trials (HR 0.26, [0.16–0.41], p<0.001, Figure 3B) and RCT (HR 0.43, [0.28–0.67], p<0.001, Figure 3B), and improved OS in both retrospective trials (HR 0.25, [0.18–0.34], p<0.001, Figure 3C) without considerable differences across trials (Heterogeneity p = 0.764, I^2^ = 0.0%, Figure 3C) and RCT (HR 0.31, [0.17–0.56], p<0.001, Figure 3C) were observed. There were also ORR benefits for BRAF WT in retrospective trials (RR 0.20, [0.08–0.52], p = 0.001, Figure 3A) with no significant variation between trials (Heterogeneity p = 0.988, I^2^ = 0.0%, Figure 3A), and a benefit in RCTs (RR 0.38 [0.20–0.73], p = 0.004, Figure 3A).

## Discussion

We performed the meta-analysis for the prognostic effects of anti-EGFR moAbs on mCRC patients with WT or mutant BRAF, which were based on the results of 21 eligible trials. The overall rate of BRAF mutation (7.4%) was similar to previously reported series [48]. The results demonstrated that patients with BRAF WT had decreased risks of progression (PFS: HR 0.38, p<0.001) and death (OS: HR 0.35, p<0.001) than patients with BRAF mutant. However, evidence of increased response in patients with BRAF WT was not enough (p = 0.328) comparing to BRAF mutated patients. In the subgroup analysis of different study types, there were still benefits for PFS and OS, but also not enough evidence of a response benefit for BRAF WT patients. In KRAS WT patients, results showed patients with BRAF WT not only decreased the risks of progression (PFS: HR 0.29, p<0.001) and death (OS: HR 0.26, p<0.001), but also increased responses (p<0.001) over these with BRAF mutant. Results were still consisted in the subgoup analysis of differents study types. Differences decreased in subgroup analysis and the conclusions didn’t change in the sensitivity analysis.

Although a previous meta-analysis from Mao C, et al [13] demonstrates larger response benefit of anti-EGFR MoAbs in BRAF WT patients over BRAF mutant patients, they did not compare other indicators such as PFS and OS. Another published meta-analysis based on the OPUS and CRYSTAL trials from Bokemeyer C, et al, [12] indicates that adding Cetuximab to chemotherapy in mCRC is beneficial for BRAF WT patients, not for BRAF mutant patients. However, the study involves only two RCTs and is conducted only in KRAS WT patients. Direct comparison between BRAF mutant and BRAF WT on effects of MoAbs is also not reported. In this meta-analysis, we made direct comparison on effects of MoAbs between patients with mutant and WT BRAF. Generally speaking, our results confirmed that mCRC patients with BRAF mutant treated with MoAbs have poorer prognosis than these with BRAF WT, especially in KRAS WT population.

We know the limitations of our meta-analysis. Firstly, retrospective trials were also included, which may cause selective bias. Secondly, only four trials reported HRs and variances as we wanted. We had to calculate or convert HRs and variances for other trials from reported survival curves, which may introduce unavoidable bias. Thirdly, size effects from retrospective and prospectived trials are unadjusted, whilst size effects from RCTs are adjusted by patient baseline characteristics. Because individual patient data was not available, we conducted meta-analysis based on unadjusted and adjusted estimations,which may introduce confounding bias. Finally, the first and second end points were incosistant across different trials, so we didn’t define them in this review.

Despite these limitations above, we confirm the conclusion that BRAF mutant is a predictive biomarker for poor prognosis in mCRC patients receiving anti-EGFR MoAbs therapy, especially in patients with KRAS WT. Therefore, screening for BRAF WT may promote the selection of potential mCRC patients whom will benefit from anti-EGFR moAbs.

## Supporting Information

Figure S1 **Begg’s funnel plot of ORR in BRAF mutant patients over these with BRAF wild-type (Egger's test: p = 0.481) in unselected patients.**
(TIF)

Figure S2 **Begg’s funnel plot of PFS in BRAF mutant patients over these with BRAF wild-type (Egger's test: p = 0.185) in unselected patients.**
(TIF)

Figure S3 **Begg’s funnel plot of OS in BRAF mutant patients over these with BRAF wild-type (Egger's test: p = 0.691) in unselected patients.**
(TIF)

Table S1 **Assessment of study quality.**
(DOC)

Table S2 **Sensitivity analysis to explore the heterogeneity between studies.**
(DOC)
