# Supplementary figures and images for: The Prognostic Role of BRAF Mutation in Metastatic Colorectal Cancer Receiving Anti-EGFR Monoclonal Antibodies: A Meta-Analysis
Source: PLoS One. 2013 Jun 11;8(6):e65995. doi: 10.1371/journal.pone.0065995 (PMC3679027; doi:10.1371/journal.pone.0065995)

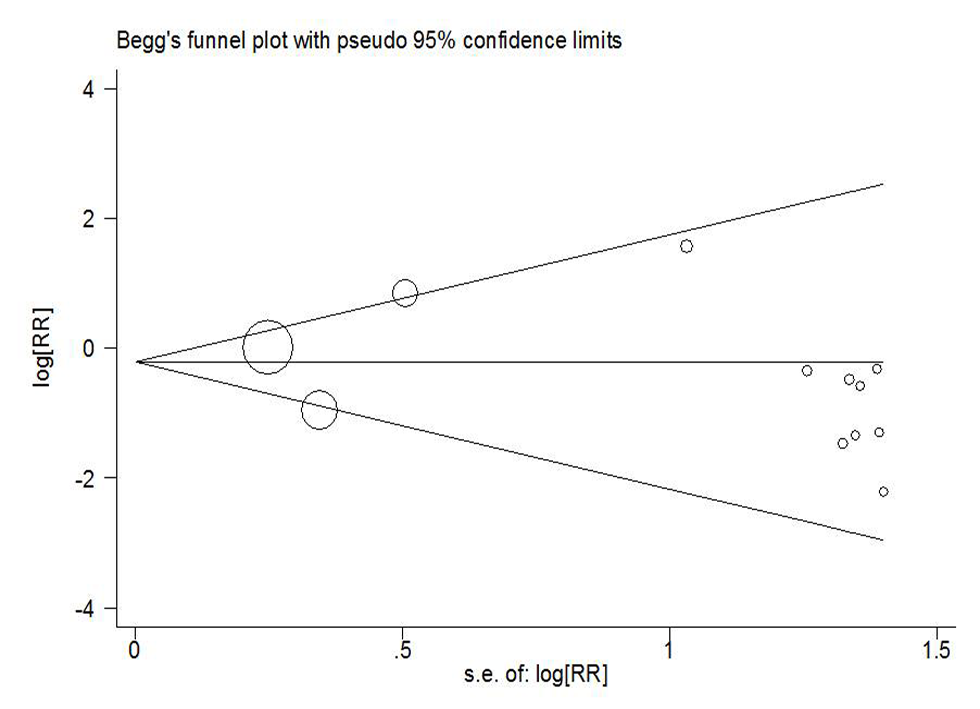

Supplement: Figure S1 — Begg’s funnel plot of ORR in BRAF mutant patients over these with BRAF wild-type (Egger's test: p = 0.481) in unselected patients. (TIF) [file pone.0065995.s001.tif]

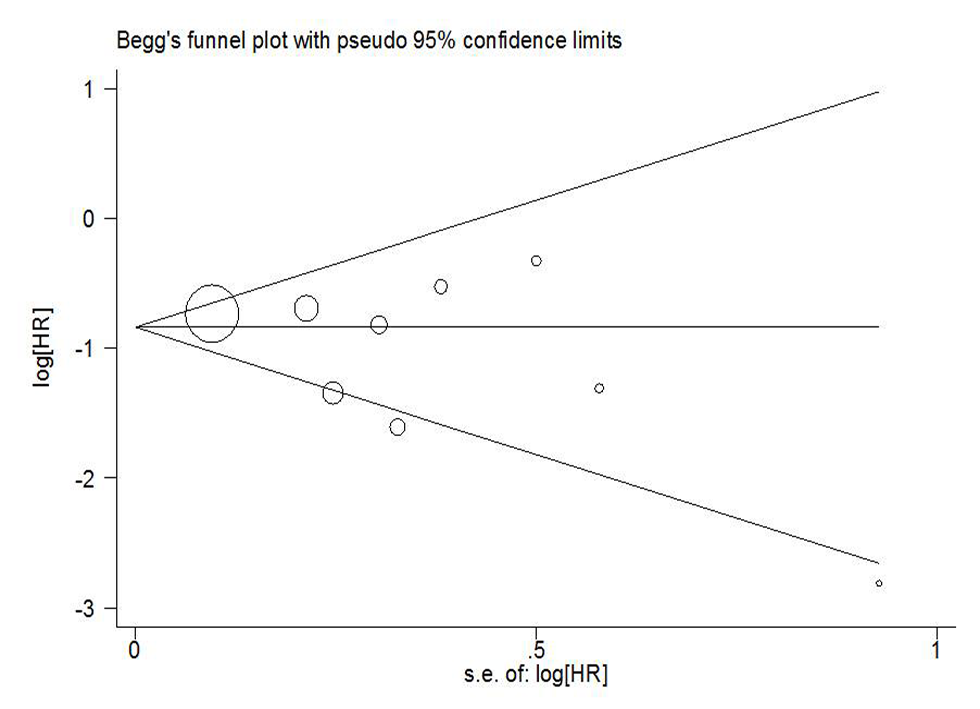

Supplement: Figure S2 — Begg’s funnel plot of PFS in BRAF mutant patients over these with BRAF wild-type (Egger's test: p = 0.185) in unselected patients. (TIF) [file pone.0065995.s002.tif]

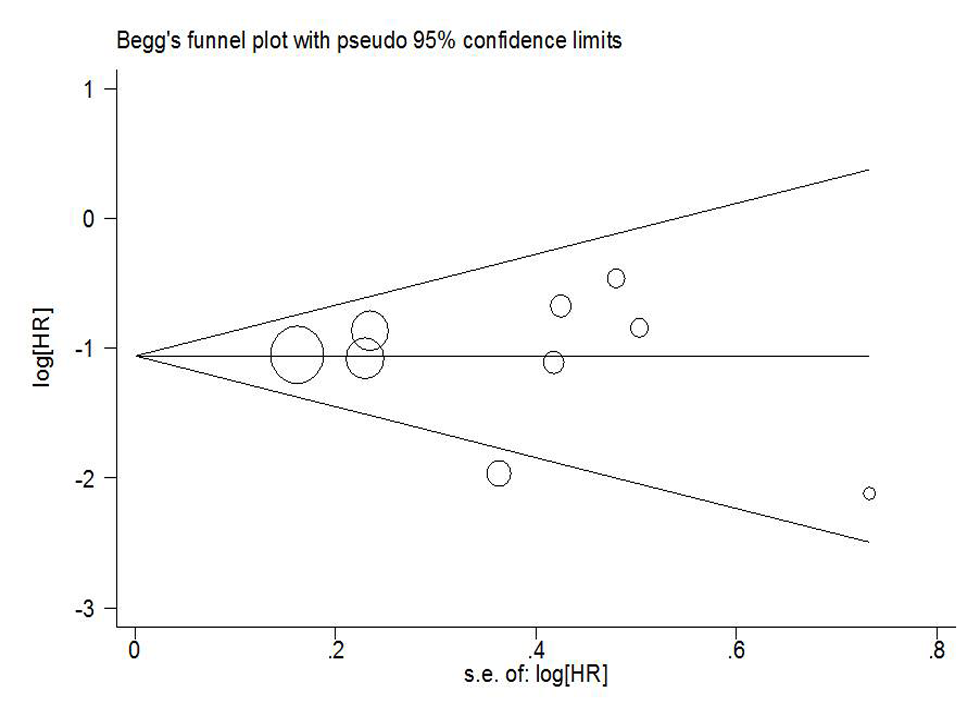

Supplement: Figure S3 — Begg’s funnel plot of OS in BRAF mutant patients over these with BRAF wild-type (Egger's test: p = 0.691) in unselected patients. (TIF) [file pone.0065995.s003.tif]
