# Supplementary material for: The Prognostic Role of BRAF Mutation in Metastatic Colorectal Cancer Receiving Anti-EGFR Monoclonal Antibodies: A Meta-Analysis
Source: PLoS One. 2013 Jun 11;8(6):e65995. doi: 10.1371/journal.pone.0065995 (PMC3679027; doi:10.1371/journal.pone.0065995)
Supplement: Table S1 — Assessment of study quality. (DOC) [file pone.0065995.s004.doc]

**Table S1: Assessment Quality**

(Based on Hayden., et al. 2006 and Z.N.Maan., et al. 2012)

| **Potential Bias** | **Items To Be Considered for Assessment of Potential Opportunity for Bias** |
| --- | --- |
| **Study participation** | |
| | Yes Partly No Unsure | | --- | | | | | | The population of interest is adequately described for key characteristics. The sampling method, period of recruitment and place of recruitment are adequately described.  Inclusion and exclusion criteria are adequately described.  The baseline study sample is adequately described for key characteristics. | | --- | | | | |
| **Study attrition** | |
| | Yes Partly No Unsure | | --- | | | | | | Proportion of study sample completing the study and providing outcome data is adequate. Attempts to collect information on participants who dropped out of the study are described.  Reasons for loss to follow-up are provided. Participants lost to follow-up are adequately described for key characteristics.  There are no important differences between key characteristics and outcomes in participants who completed the study and those who did not. | | --- | | | | |
| **Preditor measurement** | |
| | Yes Partly No Unsure | | --- | | | | | A clear definition or description of the predictors measured is provided. The predictors’ measure and method are adequately valid and reliable to limit misclassification bias. Adequate proportion of the study sample has complete data for predictors.  The method and setting of measurement are the same for all study participants. |
| **Outcome measurement** | |
| | Yes Partly No Unsure | | --- | | | | | | A clear definition of the outcome of interest is provided. The outcome measure and method used are adequately valid and reliable to limit misclassification bias. The method and setting of measurement are the same for all study participants. | | --- | | | | |
| **Confounding measurement and account** | |
| | Yes Partly No Unsure | | --- | | | | | | All important confounders are clearly defined and measured. The measurement is valid and reliable. The method and setting of confounding measurement are the same for all study participants. Important potential confounders are accounted for in the study design. Important potential confounders are accounted for in the analysis. | | --- | | | | |
| **Analysis** | |
| | Yes Partly No Unsure | | --- | | | | | | There is sufficient presentation of data to assess the adequacy of the analysis. The strategy for inclusion of variables is appropriate and is based on a conceptual framework or model. The selected method of analysis is adequate for the design of the study. There is no selective reporting of results. | | --- | | | | |

**Quality Score**

| **Components** | **Score (Yes =2, Partly = 1, No/Unsure = 0)** |
| --- | --- |
| Study Participation | 2 |
| Study Attrition | 2 |
| Predictor Measurement | 2 |
| Outcome Measurement | 2 |
| Confounding Measurement and Account | 2 |
| Analysis | 2 |
| **Total** | 12 |

**References**

1. Hayden JA, Cote P, Bombardier C (2006) Evaluation of the quality of prognosis studies in systematic reviews. Ann Intern Med 144: 427-437.

2. Maan ZN, Maan IN, Darzi AW, Aggarwal R (2012) Systematic review of predictors of surgical performance. Br J Surg 99: 1610-1621.
