# Supplementary material for: The Prognostic Role of BRAF Mutation in Metastatic Colorectal Cancer Receiving Anti-EGFR Monoclonal Antibodies: A Meta-Analysis
Source: PLoS One. 2013 Jun 11;8(6):e65995. doi: 10.1371/journal.pone.0065995 (PMC3679027; doi:10.1371/journal.pone.0065995)
Supplement: Table S2 — Sensitivity analysis to explore the heterogeneity between studies. (DOC) [file pone.0065995.s005.doc]

| Meta-analysis | Studies enrolled | Outliers | Outcome of sensitivity analysis |
| --- | --- | --- | --- |
| RR in unselected patients(RCTs) | Tveit(2011)  Modest(2012) | Outlier: n/a(2 studies only)  Heterogeneity: I^2^ =81.1%  Suspected reason: Based on investigation of study characteristics, heterogeneity could not be explained. | As no reason for heterogeneity was suspected, heterogeneity was accepted. |
| PFS in KRAS wild-type patietns (retrospective studies) | De Roock(2010)  Di Nicolantonio(2008)  Laurent-Puig(2009)  Park(2011)  Saridaki(2011)  Sartore-Bianchi(2009) | Outlier: none  Heterogeneity: I^2^ =53.0%  Suspected reason: Based on investigation of study characteristics, heterogeneity could not be explained. | As no reason for heterogeneity was suspected, heterogeneity was accepted. |

**Table S2: Sensitivity analysis to explore the heterogeneity between studies.**

Abbreviations: n/a, not applicable; RR, Risk Ratio; PFS, Progression Free Survival.

**Reference**

1. Flenady V, Koopmans L, Middleton P, Froen JF, Smith GC, et al. (2011) Major risk factors for stillbirth in high-income countries: a systematic review and meta-analysis. Lancet 377: 1331-1340.
